# A *COL11A2* Mutation in Labrador Retrievers with Mild Disproportionate Dwarfism

**DOI:** 10.1371/journal.pone.0060149

**Published:** 2013-03-20

**Authors:** Mirjam Frischknecht, Helena Niehof-Oellers, Vidhya Jagannathan, Marta Owczarek-Lipska, Cord Drögemüller, Elisabeth Dietschi, Gaudenz Dolf, Bernd Tellhelm, Johann Lang, Katriina Tiira, Hannes Lohi, Tosso Leeb

**Affiliations:** 1 Institute of Genetics, Vetsuisse Faculty, University of Bern, Bern, Switzerland; 2 Düsseldorf, Germany; 3 Department of Veterinary Clinical Science, Small Animal Clinic, Justus-Liebig-University, Giessen, Germany; 4 Department of Clinical Veterinary Medicine, Division of Clinical Radiology, Vetsuisse Faculty, University of Bern, Bern, Switzerland; 5 Research Programs Unit, Molecular Medicine, University of Helsinki, Helsinki, Finland; 6 Department of Veterinary Biosciences and Department of Medical Genetics, University of Helsinki, Helsinki, Finland; 7 Folkhälsan Institute of Genetics, Helsinki, Finland; University of Sydney, United States of America

## Abstract

We describe a mild form of disproportionate dwarfism in Labrador Retrievers, which is not associated with any obvious health problems such as secondary arthrosis. We designate this phenotype as skeletal dysplasia 2 (SD2). It is inherited as a monogenic autosomal recessive trait with incomplete penetrance primarily in working lines of the Labrador Retriever breed. Using 23 cases and 37 controls we mapped the causative mutation by genome-wide association and homozygosity mapping to a 4.44 Mb interval on chromosome 12. We re-sequenced the genome of one affected dog at 30x coverage and detected 92 non-synonymous variants in the critical interval. Only two of these variants, located in the lymphotoxin A (*LTA*) and collagen alpha-2(XI) chain gene (*COL11A2*), respectively, were perfectly associated with the trait. Previously described *COL11A2* variants in humans or mice lead to skeletal dysplasias and/or deafness. The dog variant associated with disproportionate dwarfism, *COL11A2:c.143G>C* or p.R48P, probably has only a minor effect on collagen XI function, which might explain the comparatively mild phenotype seen in our study. The identification of this candidate causative mutation thus widens the known phenotypic spectrum of *COL11A2* mutations. We speculate that non-pathogenic *COL11A2* variants might even contribute to the heritable variation in height.

## Introduction

Height is a textbook example for a complex trait with high heritability and polygenic inheritance [1]. In humans, more than 180 loci have been identified, which contribute to the normal variation in height [2]. In humans the influence of each of these loci is relatively small. Currently, even the largest genome-wide association studies (GWASs) with ∼180,000 probands can only explain a limited fraction of the observed genetic variance in human height [2]. One possible explanation for the so-called “missing heritability” in human height studies is the presence of rare variants with small or intermediate effects [3]. If the allele frequency of such variants is low, they would not be expected to be detected in GWAS studies. On the other hand, if their phenotypic effect is not quite as large as to raise suspicion of a Mendelian phenotype, individuals carrying such rare variants may remain undetected.

In comparison to humans, domestic animals offer a more favorable population structure with reduced heterogeneity within isolated breeds, which facilitates the identification of loci influencing height [4], [5]. Recently, several studies in cattle, dogs, and horses have shown that in these animals a small number of genes with relatively large effects are major determinants of height and body size [6]–[11].

It is becoming increasingly clear that there is a more or less continuous overlap between the polygenic natural variation in height and phenotypes caused by single genes with relatively large effects on skeletal development [3]. Many of these Mendelian traits result in very severe deviations from a normal stature or are associated with symptoms in other organ systems, so that they can be readily recognized. Currently, based on radiological and clinical criteria there are more than 450 skeletal dysplasias in humans [12]. A subset of these skeletal dysplasias is caused by mutations in the genes encoding the subunits of collagen types II, IX and XI [13]. Thus, if the underlying molecular genetics of skeletal dysplasias of varying severity is solved, these Mendelian traits help to shed further light into the complex biology of skeletal growth.

In dogs several genes leading to a “short-legged phenotype” or disproportionate dwarfism have been described. The presence of a retro-transposed extra-copy of the *FGF4* gene leads to chondrodysplasia, which is a breed-defining trait in many short-legged breeds such as e.g. the Dachshund and Basset Hound [14]. In the Labrador Retriever a heritable phenotype involving short legs and severe ocular defects, termed oculoskeletal dysplasia (OSD) is known to be caused by a mutation in the *COL9A3* gene encoding the α3-chain of collagen type IX [15]. Another chondrodysplastic phenotype in Labrador Retrievers with presumed monogenic autosomal recessive inheritance is characterized by short curved legs (radius curvus deformity) leading to secondary joint problems such as arthrosis. This type of chondrodysplasia in Labrador Retrievers is not associated with eye defects and its causative mutation has not yet been reported [16].

We report here the phenotypic and genetic analysis of another distinct short-legged phenotype termed skeletal dysplasia 2 (SD2) in Labrador Retrievers, which has a very subtle phenotype without any obvious ocular or auditory involvement and without known secondary joint problems.

## Results

### Phenotypic description

**Figure 1:**
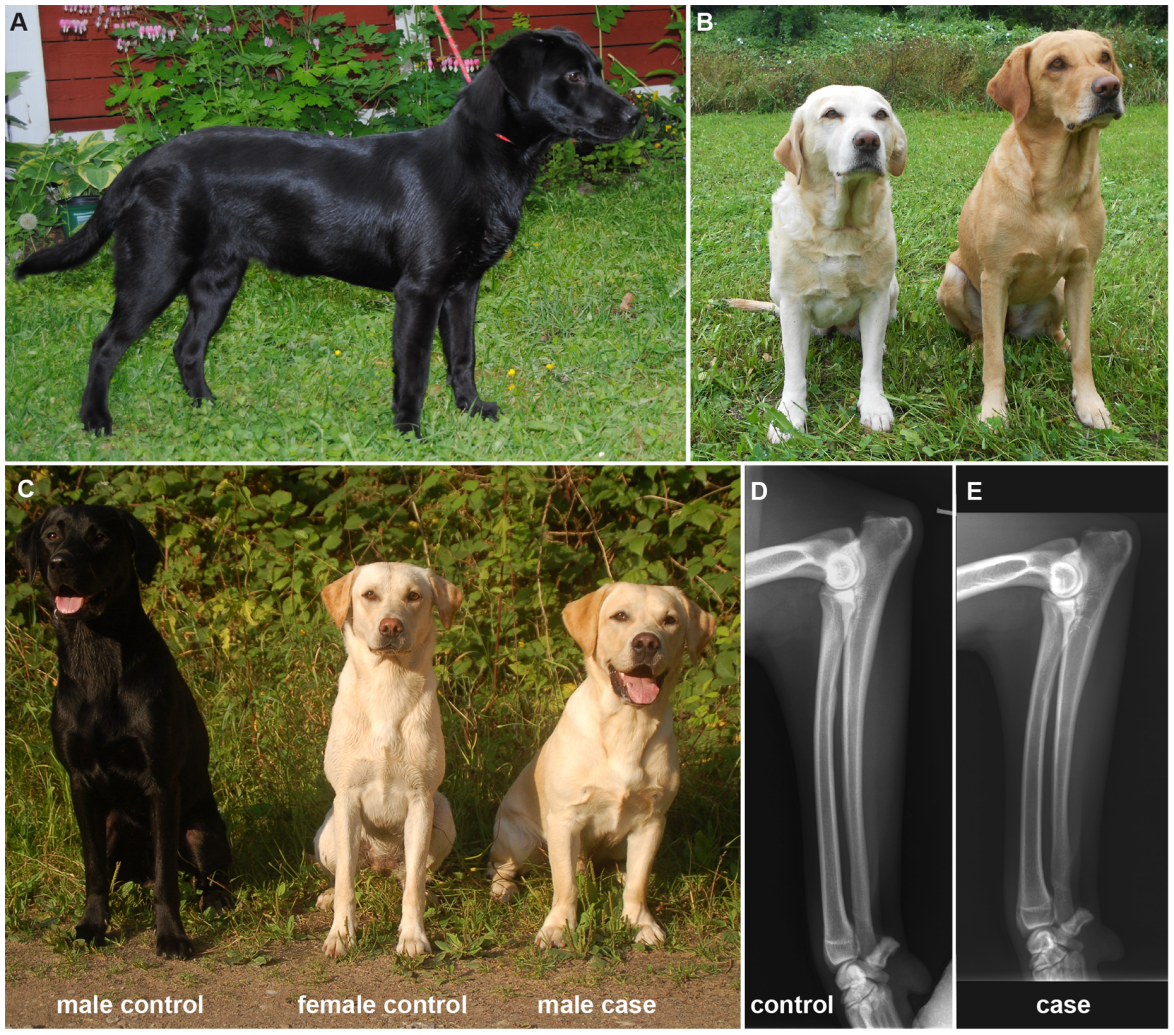
Phenotype of the inherited disproportionate dwarfism. (A) Photograph of a female affected Labrador Retriever. Notice the relatively long body in relation to the length of the legs. (B) Affected mother (left) with her non-affected daughter (right). The affected dog has shorter forelegs. (C) Photograph of three littermates. The affected male has short legs and different proportions compared to his non-affected sister. (D) Radiograph of the left front limb of a male control Labrador Retriever and (E) his affected full brother at 12 months of age. In the affected dog ulna and radius are 2.7 cm and 1.9 cm shorter and slightly more bent than in the control, respectively. The diameter of the diaphysis of the long bones is not affected (Table S1).

In the Labrador Retriever breed, a hereditary form of mild disproportionate dwarfism was recognized by the breeders, which we termed SD2 to distinguish it from OSD. We obtained information on 34 affected dogs. Their phenotype was characterized by short legs with normal body length and width (Figure 1A). In most cases the forelegs were slightly more affected than the hind legs. Male affected dogs mostly had shoulder heights of less than 55 cm and female affected dogs mostly had shoulder heights of less than 50 cm, while the international breed standards calls for 56 cm–57 cm in males and 54 cm–56 cm in females respectively (www.fci.be). However, the height of affected dogs is quite variable and there is an overlap in shoulder height between small non-affected Labrador Retrievers and tall Labrador Retrievers, which are affected by this particular form of mild disproportionate dwarfism. Thus, our phenotypic classification depended on the measurement of the shoulder height in combination with an evaluation of the overall proportions of the dogs. We encountered several dogs, which we could not unambiguously classify as affected or non-affected. The ambiguous dogs were excluded from the further study. The owners of affected dogs did not report signs of degenerative joint disease in their dogs. This is consistent with our radiological findings in one affected dog. This dog had slightly shortened long bones with relatively wide epiphyses. We did not observe any major alterations in the joints (Figure 1E; Table S1).

### Pedigree analysis

**Figure 2:**
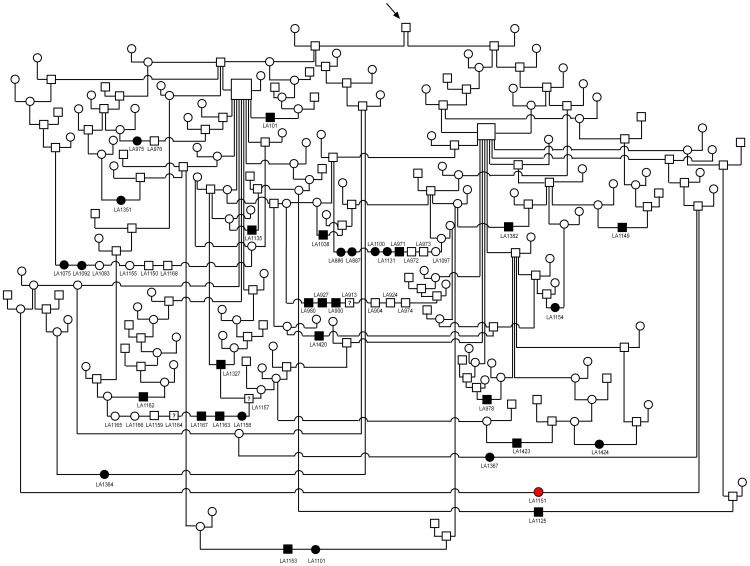
Pedigree of 33 Labrador Retrievers with mild disproportionate dwarfism. Affected dogs are shown as filled symbols together with their laboratory identifiers. One dog with severely shortened legs and deformed paws that did not carry the disease-associated haplotype is highlighted in red (see also Figure S1). Three dogs that could not unambiguously be classified are indicated with question marks. Please note that due to space restrictions many non-affected littermates of the affected dogs are not shown in this pedigree. All affected dogs in this pedigree can be traced to a common ancestor in their paternal and maternal ancestry, respectively (arrow). This male dog was born in 1966 and may represent the founder of SD2.

We obtained the pedigree information from 33 of the 34 affected individuals (Figure 2). The affected dogs came from so-called working lines as opposed to show lines within the Labrador Retriever breed. The frequency of affected dogs was equal between males and females. The pedigree data are consistent with a monogenic autosomal recessive mode of inheritance of the trait. All affected dogs in our study are related to each other and trace back to a male Labrador Retriever born in 1966. This dog was a successful working dog and consequently became a popular sire who contributed to the spreading of SD2 in the population. This dog might represent the actual founder of the causative mutation event. In this case, the mutation would have occurred less than 10 generations ago.

### Mapping of the causative mutation

**Figure 3:**
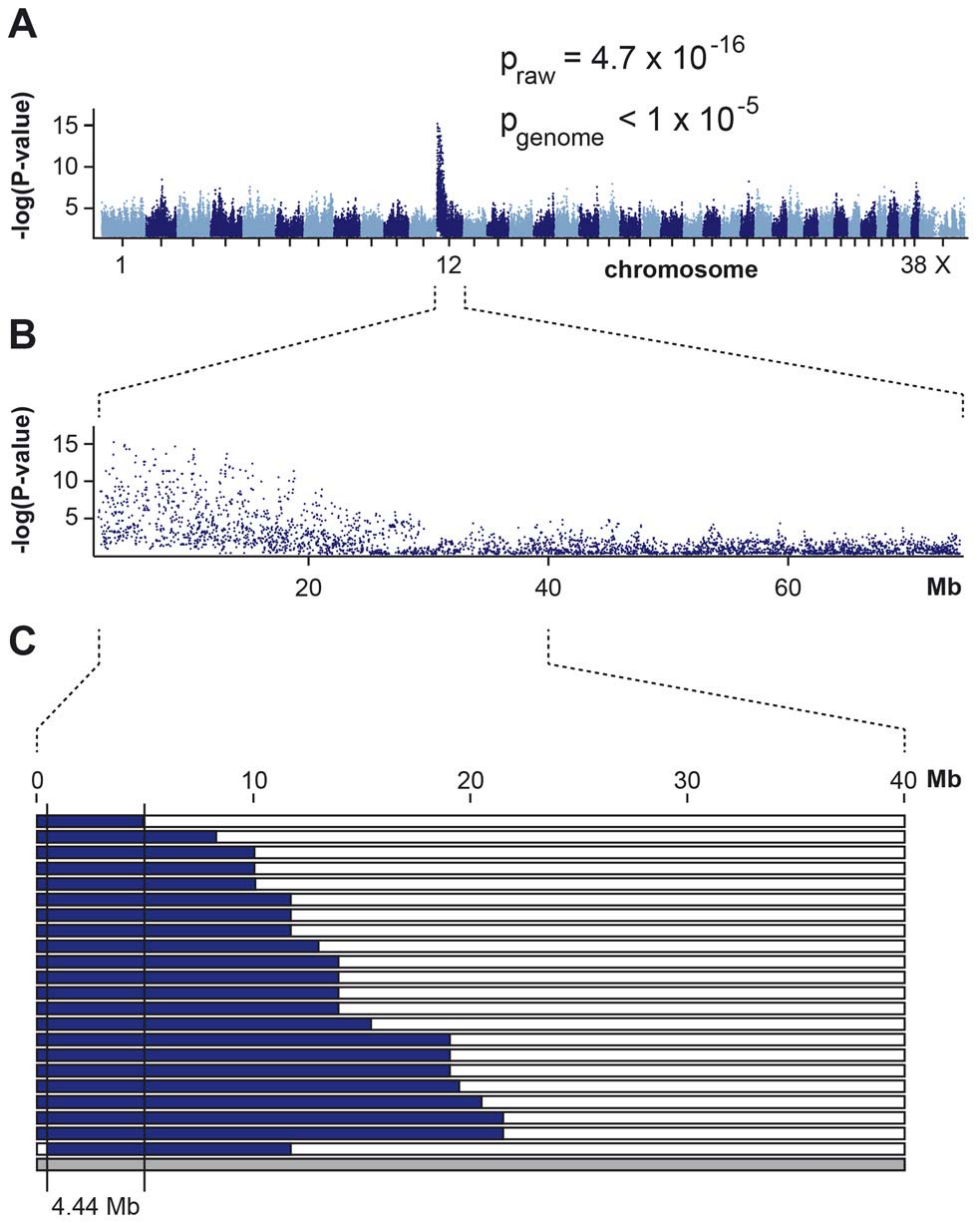
Mapping of SD2 in Labrador Retrievers. (A) A genome-wide association study using 23 cases and 37 controls indicates a strong signal with multiple associated SNPs on CFA 12. (B) The detailed view of CFA 12 delineates an associated interval of ∼18 Mb at the beginning of the chromosome. (C) Homozygosity mapping. Each horizontal bar corresponds to one of the 23 analyzed cases. Homozygous regions with shared alleles are shown in blue. A shared homozygous interval of ∼4 Mb in 22 of the 23 cases delineates the exact boundaries of the critical interval from 467,795 bp to 4,906,914 bp (CanFam 3 assembly). The 23^rd^ case did not carry the associated haplotype and was also phenotypically slightly distinct from the other 22 cases (Figure S1).

We genotyped 173,662 evenly spaced SNPs on DNA samples from 23 affected Labrador Retrievers and 37 controls. After removing 54,161 markers, which had bad call rates (<90%), were non-informative (MAF <0.05), or showed a strong deviation from Hardy-Weinberg equilibrium in the controls (p<10^−5^), we retained 119,501 markers for the final genome-wide allelic association study. The best-associated SNP in the GWAS had a raw p-value of 4.7×10^−16^ (Figure 3A). The corrected p-value after 100,000 permutations was <10^−5^. It has to be cautioned that the genomic inflation factor in this analysis was 2.75. This extremely high value was caused by two factors: (1) to a large extent by the use of highly stratified and sometimes closely related samples and (2) to a small extent the fact that a relatively large portion of the genome showed an association (Figure S2). The 169 best-associated SNPs with raw p-values of less than 1.7×10^−8^ were all located at the beginning of CFA 12 (Figure 3B). These GWAS results showed an unequivocal signal and were compatible with a common, relatively young causative mutation for a monogenic trait. We also performed a mixed-model analysis controlling for the population stratification, which showed the most significant association at the same marker as before (Pc1df  = 1.83×10^−8^).

Subsequently, we applied a homozygosity mapping approach to fine-map the region containing the SD2 mutation. Based on the pedigree records we hypothesized that the affected dogs most likely were inbred to one single founder animal (Figure 2). Under this scenario the affected individuals were expected to be identical by descent (IBD) for the causative mutation and flanking chromosomal segments. We analyzed the cases for extended regions of homozygosity with simultaneous allele sharing. Only one genome region, which coincided with the associated interval on CFA 12, fulfilled our search criteria. Here, 22 out of 23 affected dogs were homozygous and shared identical alleles over 344 SNP markers corresponding to a ∼4 Mb interval. We concluded that the causative mutation should be located in the 4.44 Mb critical interval between the closest heterozygous markers on either side of the homozygous segment (CFA12: 467,795–4,906,914, CanFam 3 assembly; Figure 3C).

One of the 23 cases did not carry the associated haplotype. This dog had a very severe phenotype, which not only included very short legs, but also slightly deformed paws (Figure S1). Thus we assumed that the skeletal dysplasia in this dog was caused by a distinct genetic or environmental effect and excluded it from further analysis.

### Mutation identification

A total of 171 genes and loci are annotated in the critical interval on CFA 12 (CanFam 3.1). In the orthologous human segment on HSA 6, 232 genes and loci are annotated (human genome build 37.3). To get a comprehensive overview of all variants in the critical interval we sequenced the whole genome of one SD2 affected Labrador Retriever. We collected 900 million 100 bp paired-end reads from a standard 300 bp fragment library corresponding to roughly 30x coverage of the genome. We called SNPs and indel variants with respect to the reference genome of a presumably non-affected Boxer. Across the entire genome, we detected 2.5 million homozygous variants (Table 1). Within the critical interval there were 10,508 variants, of which 92 were predicted to be non-synonymous (Table S2). We further compared the genotypes of the affected Labrador Retriever with 13 dog genomes of various breeds that had been sequenced in our laboratory in the course of other ongoing studies. We hypothesized that the mutant allele at the causative variant should be completely absent from all other dog breeds outside the Labrador Retrievers as the pedigree analysis and the large size of the associated haplotype clearly indicated a relatively young origin of the mutation. Therefore, we considered it unlikely that the mutant allele would have been introgressed into any other breeds outside the Labrador Retrievers. Among the 92 non-synonymous variants, there were only 5 variants where the affected Labrador Retriever carried the homozygous variant genotype and all other 13 sequenced dogs carried the homozygous wildtype genotype (Table 2).

**Table 1:** Variants detected by whole genome re-sequencing of an affected Labrador Retriever.

Filtering step	Number of variants
Variants in the whole genome^a^	2,513,422
Variants in the critical 4.44 Mb interval on CFA 12	9,097
Variants in the critical interval that were absent from 13 other dog genomes	762
Non-synonymous variants in the whole genome^a^	8,146
Non-synonymous variants in the critical 4.44 Mb interval on CFA 12	92
Non-synonymous variants in the critical interval that were absent from 13 other dog genomes	5

a The sequences were compared to the reference genome (CanFam 3) from a Boxer. Only variants that were homozygous in the affected Labrador Retriever are reported.

**Table 2:** Five non-synonymous variants in the critical interval of an affected Labrador Retriever that were absent from 13 other dog genomes.

Position on CFA 12 (CanFam 3 assembly)	Reference allele	Variant allele	Gene	Variant (cDNA)	Variant (protein)
1,071,693	C	T	*LTA*	c.140C>T	p.A47V
1,589,339	C	T	*NOTCH4*	c.4774G>A	p.E1592K
2,587,404	G	A	*LOC100684308*	c.397C>T	p.P133S
2,636,467	G	A	*COL11A2*	c.3022C>T	p.P1008S
2,652,874	C	G	*COL11A2*	c.143G>C	p.R48P

We genotyped all remaining non-synonymous variants in larger cohorts of dogs (Table 3). Three of these variants could be excluded as being causative for SD2, as we identified several non-affected Labrador Retrievers carrying the non-reference alleles in homozygous state. Two variants, *COL11A2:c.143G>C* and *LTA:c140C>T* remained perfectly associated with the SD2 phenotype in more than 700 Labrador Retrievers. Both variants were also absent from a selection of dogs from other breeds (Table S3). Among the 86 presumed SD2 carriers in the unrelated Labrador Retriever controls two dogs were considered to be showline dogs.

**Table 3:** Association of non-synonymous variants with the dwarfism phenotype.

Genotype	Labrador Retriever cases	Labrador Retriever non-affected carriers^a^	Labrador Retriever controls	Dogs from other breeds
*LTA:c140C>T*				
* C/C*	-	-	620	91
* C/T*	-	12	87	-
* T/T*	13	-	-	-
*NOTCH4:c.4774G>A*				
* G/G*	-	-	60	13
* G/A*	-	6	53	-
* A/A*	11	-	7	-
*LOC100684308:c.397C>T*				
* C/C*	-	-	25	12
* C/T*	-	5	37	-
* T/T*	9	-	7	-
*COL11A2:c.3022C>T*				
* C/C*	-	-	67	12
* C/T*	-	9	50	-
* T/T*	19	1	4	-
***COL11A2:c.143G>C***				
*** G/G***	**1** b	-	**621**	**277** c
*** G/C***	-	**12**	**86**	-
*** C/C***	**33**	-	-	-

a Parents of affected dogs were classified as obligate carriers.

b This dog had a very severe dwarfism phenotype with additional deformities of the paws (Figure S1).

c A list of these 277 dogs from 83 diverse dog breeds is given in Table S2.

The *COL11A2* gene encoding the α2-chain of collagen type XI has a known function in skeletal development [17]–[19]. In contrast, the *LTA* gene encodes lymphotoxin α, a cytokine and member of the TNF-superfamily, which is secreted by T-cells [20]. Thus we concluded that the *COL11A2:c.143G>C* variant is much more likely to cause SD2 than the *LTA* variant.

**Figure 4:**
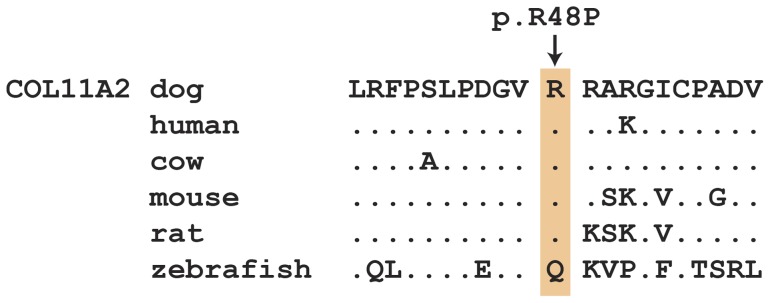
COL11A2 protein alignment. The p.R48P variant is located in a region of moderate sequence conservation between the N-terminal signal peptide (amino acids 1–27) and the laminin G-like domain (amino acids 57–228). The sequences correspond to the following accessions: XP_538855.2 (*canis familiaris*), NP_542411.2 (*homo sapiens*), NP_001039664.1 (*bos taurus*), NP_034056.1 (*mus musculus*), NP_997693.1 (*rattus norvegicus*), NP_001073461.1 (*danio rerio*).

The *COL11A2:c.143G>C* variant is predicted to result in the non-conservative exchange of a proline for an arginine (p.R48P), located in a structurally flexible region between the signal peptide and the laminin G-like domain of the collagen alpha-2(XI) precursor. *In silico* tools predicting the functional effect indicate that the p.R48P variant is tolerated (SIFT score 0.47 [21]) or benign (Polyphen-2 score 0.108 [22]). Although position 48 of the peptide chain is not within any functionally annotated domain, this region of the protein is nonetheless highly conserved among mammals (Figure 4).

### Genotype-phenotype correlation

**Figure 5:**
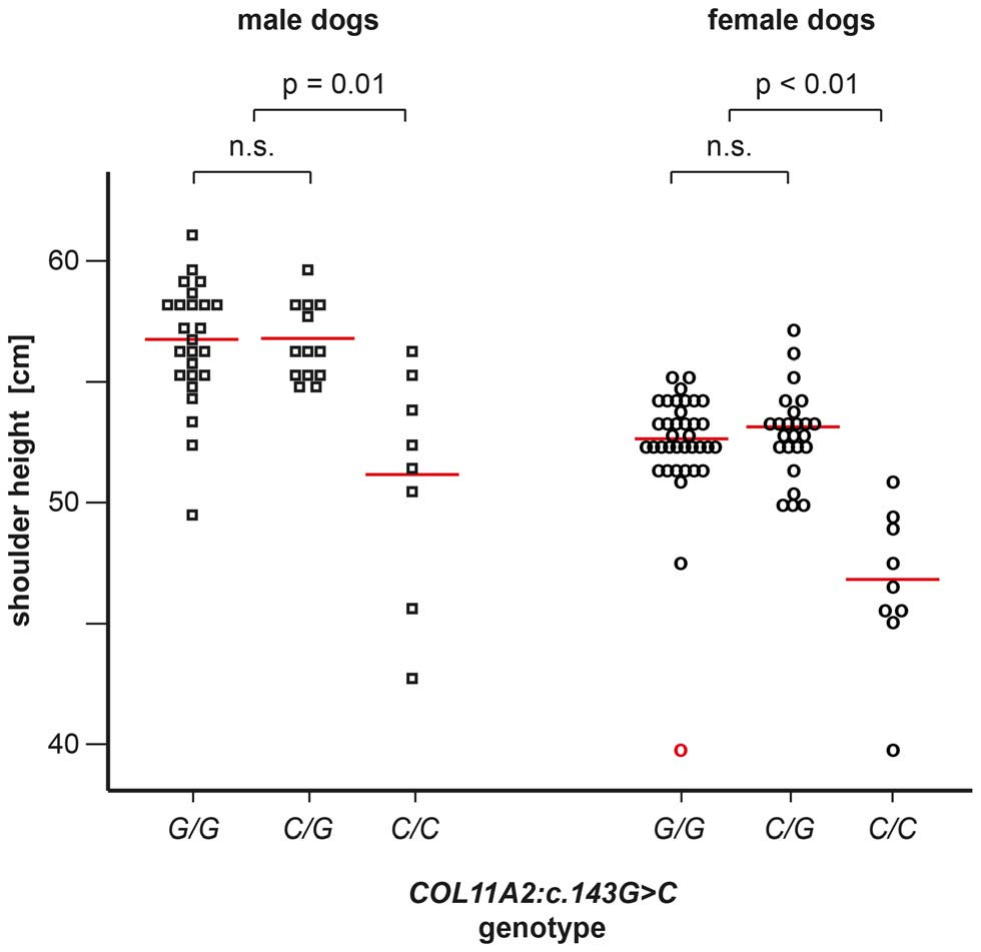
Genotype-phenotype correlation. The figure displays the shoulder heights of male and female Labrador Retrievers with the three different genotypes at the *COL11A2:c.143G>C* variant. The red lines indicate the averages of each group. The size distributions confirm that SD2 is inherited as recessive trait. The female outlier indicated in red was phenotyped as affected by disproportionate dwarfism, but did not carry the *COL11A2* variant (see also Figure 2 and Figure S1). Please note that there is a considerable overlap in the shoulder height distributions of SD2 cases and controls, reflecting the fact that height is a complex polygenic trait in dogs. We used shoulder height as a proxy for the SD2 phenotype in this analysis as it was the only quantitative phenotype that was available to us on a sufficiently large number of animals. However, dogs with the C/C phenotype typically had different proportions from dogs with the other two genotypes, even if they had a normal shoulder height.

We obtained shoulder height measurements on 130 Labrador Retrievers (Figure 5). The average shoulder height of male control Labrador Retrievers in our study was 57.4 cm, while the female controls in our dataset had an average shoulder height of 53.5 cm, respectively. We then compared the height distributions between the different genotype classes at *COL11A2:c.143G>C*. Neither the variances nor the means of the measurements of the shoulder height differed within males or within females between the genotypes G/G and C/G. Therefore, we pooled the measurements of the G/G and C/G genotypes for comparison with those of the C/C genotype.

The variances between the combined G/G and C/G genotypes versus the C/C genotypes differed in both, male (p<0.01) and female (p<0.01). The subsequent comparison of the corresponding means also clearly showed a difference in the shoulder height within males (p = 0.01) and females (p<0.01). Males with the C/C genotype were on average 5.8 cm smaller (95% CI 47.5 cm–55.6 cm) than males with the C/G or G/G genotype (95% CI 56.6 cm–58.1 cm). Females with the C/C genotype were on average 6.5 cm smaller (95% CI 44.5 cm–49.6 cm) than females with the C/G or G/G genotype (95% CI 53.1 cm–53.9 cm).

## Discussion

Using a positional candidate gene approach, we have identified the c.143G>C variant in the canine *COL11A2* gene as the most likely causative variant for an inherited mild disproportionate dwarfism in Labrador Retrievers, which we term skeletal dysplasia 2 (SD2). Due to the strictly recessive mode of inheritance, we consider a non-coding regulatory variant unlikely to cause the observed phenotype. We have systematically excluded all other non-synonymous variants in the known genes of the critical interval with the exception of *LTA:c140C>T*, which affects a gene of the immune system, that is unlikely to play a role in bone development.

The *COL11A2* gene represents a good functional candidate gene for a skeletal dysplasia phenotype. Depending on the specific variant, mutations in the human *COL11A2* gene cause Stickler syndrome type 3 (STL3, MIM183840, [23]), otospondylomegaepiphyseal dysplasia (OSMED, MIM215150, [24]), Weissenbacher-Zweymueller syndrome (WZS, MIM277610, [25]), fibrochondrogenesis type 2 (FBCG2, MIM614524 [21]), or isolated deafness (DFNA13, MIM601868, [26] and DFNB53, MIM609706, [27]). STL3, OSMED, and WZS are inherited skeletal dysplasias of moderate severities that are associated with deafness. FBCG2 is a very severe and often lethal skeletal dysplasia, which is associated with high myopia and mild to moderate hearing loss. The milder human skeletal dysplasias STL3, OSMED and WZS share broad epiphyses as a clinical feature with SD2 in dogs from our study. Nonetheless, the skeletal aberrations of Labrador Retrievers with SD2 are very mild in comparison to all three human diseases and there are no reports of secondary joint problems in these dogs. *Col11a2* deficient mice are smaller than wildtype littermates and also show a skeletal dysplasia with hearing impairment [17]. Collagen XI nucleates the self-assembly and limits the lateral growth of cartilage fibrils, which are composed of the collagens II, IX, and XI [18]. These extracellular cartilage fibrils are required for the normal differentiation and spatial organization of growth plate chondrocytes [17], [19]. Thus, although we have no functional proof for the causality of the COL11A2:p.R48P variant and cannot formally rule out that it is a neutral variant in linkage disequilibrium with an unknown causative variant, our genetic data together with the evolutionary conservation in mammals and the functional knowledge from other species about the critical role of the *COL11A2* gene in bone growth very strongly suggest that it is indeed the causative mutation.

We have no data on deafness in our affected Labrador Retrievers. Unfortunately, it is very difficult to perform quantitative measurements of the auditory capacity in dogs. However, as these dogs are used in hunting and field trials, we assume that they do not have any severe hearing loss.

Our data indicate that COL11A2:p.R48P most likely has only a minor effect on collagen XI function. This is consistent with the location of this variant at the N-terminus, before the first functionally important protein domain of the collagen alpha-2(XI) chain. So far, all previous data on genotype-phenotype correlations for *COL11A2* variants suggested that functional variations in this gene lead to either moderate to severe deafness and/or moderate to severe skeletal dysplasias. Our results with the new dog variant indicate that the phenotypic spectrum of *COL11A2* variants may also include a very mild skeletal dysplasia without any severe hearing impairment. Thus it will be interesting to see whether there are also non-pathogenic *COL11A2* variants, which just modulate the height of an organism.

Our results enable genetic testing and the eradication of this mild form of disproportionate dwarfism from the Labrador Retriever population. However, the SD2 mutation in this study does not explain the reported inherited chondrodysplasia in Labrador Retrievers [16]. The chondrodysplasia has a more severe phenotype with secondary joint problems and the *COL11A2* gene has already previously been excluded to be causative for this phenotype [16].

In summary, we describe a new inherited form of mild disproportionate dwarfism in Labrador Retrievers and provide a non-synonymous variant in the *COL11A2* gene as candidate causative mutation. The relatively mild phenotype in the affected dogs expands the known genotype-phenotype correlations for *COL11A2* mutants and sheds further light in the complex regulation of skeletal growth.

## Materials and Methods

### Ethics statement

All animal experiments were performed according to the local regulations. The dogs in this study were examined with the consent of their owners. The study was approved by the “Cantonal Committee For Animal Experiments” (Canton of Bern; permits 22/07 and 23/10).

### Animal selection

We collected EDTA blood samples from Labrador Retrievers. We specifically used 34 cases and 147 controls, which could be unambiguously phenotyped based on photographs or direct inspection. Additionally we used Labrador Retriever DNA samples that were collected for the LUPA atopic dermatitis cohort [28] and other DNA samples that were collected for various research projects at the Institute of Genetics. For all of these samples a non-affected phenotype was assumed (“population controls”) as the investigated form of mild disproportionate dwarfism (SD2) is a young and relatively rare trait.

### DNA samples and SNP genotyping

We isolated genomic DNA from EDTA blood samples with the Nucleon Bacc2 kit (GE Healthcare). Genotyping was done on illumina canine_HD chips containing 173,662 SNP markers either at the Centre National de Génotypage, Evry, France or at the NCCR Genomics Platform of the University of Geneva. Genotypes were stored in a BC/Gene database version 3.5 (BC/Platforms).

### Genome-wide association study (GWAS) and homozygosity mapping

We used PLINK v1.07 [29] to perform genome-wide association analyses (GWAS). We removed markers and individuals with call rates <90% from the analysis. We also removed markers with minor allele frequency (MAF) <5% and markers strongly deviating from Hardy-Weinberg equilibrium (p<10^−5^). We performed an allelic association study and determined an empirical significance threshold by performing 100,000 permutations of the dataset with arbitrarily assigned phenotypes. We also used PLINK to search for extended intervals of homozygosity with shared alleles as described previously [30].

We used GenABEL [31] to perform a GWAS with correction for the population stratification. We performed an allelic association study using the function polygenic_hglm, a mixed-model approach, which uses the kinship matrix estimated from the marker data to correct for population stratification.

### Gene analysis

We used the dog CanFam 3 and the human 37 assemblies for all analyses. We used BLASTN searches to define the orthologous human chromosomal regions corresponding to the associated interval on CFA 12. For the candidate gene inspection we used the human annotation provided by NCBI (build 37.3). All numbering within the canine *COL11A2* gene corresponds to the accessions XM_538855.2 (mRNA) and XP_538855.2 (protein).

### Whole genome sequencing of an affected Labrador Retriever

We prepared a fragment library with 300 bp insert size and collected three lanes of illumina HiSeq2000 paired-end reads (2×100 bp). We obtained a total of 905,025,144 paired-end reads or roughly 30x coverage. We mapped the reads to the dog reference genome using with the Burrows-Wheeler Aligner (BWA) version 0.5.9-r16 [32] with default settings and obtained 438,768,718 uniquely mapping reads. After sorting the mapped reads by the coordinates of the sequence and merging the 3 lanes of data with Picard tools, we labeled the PCR duplicates also with Picard tools (http://sourceforge.net/projects/picard/). We used the Genome Analysis Tool Kit (GATK version 0591, [33]) to perform local realignment and to produce a cleaned BAM file. Variants calls were then made with the unified genotyper module of GATK. Variant data for each sample were obtained in variant call format (version 4.0) as raw calls for all samples and sites flagged using the variant filtration module of GATK. Variant calls that failed to pass the following filters were labeled accordingly in the call set: (i) Hard to Validate MQ0 ≥4 & ((MQ0/(1.0 * DP)) >0.1); (ii) strand bias (low Quality scores)QUAL <30.0 || (Quality by depth) QD <5.0 || (homopolymer runs) HRun >5 || (strand bias) SB >0.00; (iii) SNP cluster window size 10. The snpEFF software [34] together with the CanFam 3.1 annotation was used to predict the functional effects of detected variants. We considered the following snpEFF categories of variants as non-synonymous: NON_SYNONYMOUS_CODING, CODON_DELETION, CODON_INSERTION, CODON_CHANGE_PLUS_CODON_DELETION, CODON_CHANGE_PLUS_CODON_INSERTION, FRAME_SHIFT, EXON_DELETED, START_GAINED, START_LOST, STOP_GAINED, STOP_LOST, SPLICE_SITE_ACCEPTOR, SPLICE_SITE_DONOR. The critical interval contained 4,439,120 bp and 194,823 coding nucleotides, respectively. In our re-sequencing data, we had ≥4x coverage on 4,322,636 bp of the critical interval (97.4%) and 190,467 of the coding bases (97.8%), respectively.

Additionally, we analyzed the BAM file for large structural variants with Pindel [35]. Pindel did not detect any large structural rearrangements larger than 30 bp in the critical interval (insertions, inversions, and tandem duplications).

### Sanger sequencing

We used Sanger sequencing to confirm the illumina sequencing results and to perform targeted genotyping for selected variants. We sequenced all 66 exons of the *COL11A2* gene in two SD2 cases and two controls to exclude any structural variants affecting the coding sequence of *COL11A2*. For these experiments we amplified PCR products using AmpliTaqGold360Mastermix (Applied Biosystems). PCR products were directly sequenced on an ABI 3730 capillary sequencer (Applied Biosystems) after treatment with exonuclease I and shrimp alkaline phosphatase. We analyzed the sequence data with Sequencher 4.9 (GeneCodes).

### Statistical analysis

We obtained shoulder height measurements from 130 Labrador Retrievers. We compared the variances and means of the different genotype × gender groups using the TTEST procedure of SAS 9.3 (SAS Institute Inc., Cary, NC, USA). The variances of the measurements of the shoulder height of the genotypes G/G and C/G did not differ within males (p = 0.09) nor within females (p = 0.27). The corresponding comparisons of the means within males (p = 0.96) and within females (p = 0.74) also did not reveal any differences. Therefore, we pooled the measurements of the G/G and C/G genotypes for comparison with those of the C/C genotype.

## Supporting Information

Figure S1 Photographs of a Labrador Retriever with very short legs and deformed paws and carrying the wildtype genotype at *COL11A2:c.143G>C*.
(PDF)

Figure S2 Quantile-quantile (QQ) plot of the GWAS.
(TIF)

Table S1 Measurements of long bone dimensions in a male SD2 affected Labrador Retriever and his non-affected brother.
(XLSX)

Table S2 Non-synonymous variants in the critical interval CFA12: 467,795–4,906,914.
(XLSX)

Table S3 List of 277 dogs from 83 diverse breeds, which were free of the mutant allele at *COL11A2:c.143G>C*.
(XLSX)
