# Supplementary material for: A COL11A2 Mutation in Labrador Retrievers with Mild Disproportionate Dwarfism
Source: PLoS One. 2013 Mar 20;8(3):e60149. doi: 10.1371/journal.pone.0060149 (PMC3603880; doi:10.1371/journal.pone.0060149)
Supplement: Figure S1 — Photographs of a Labrador Retriever with very short legs and deformed paws and carrying the wildtype genotype at COL11A2:c.143G>C. (PDF) [file pone.0060149.s001.pdf]

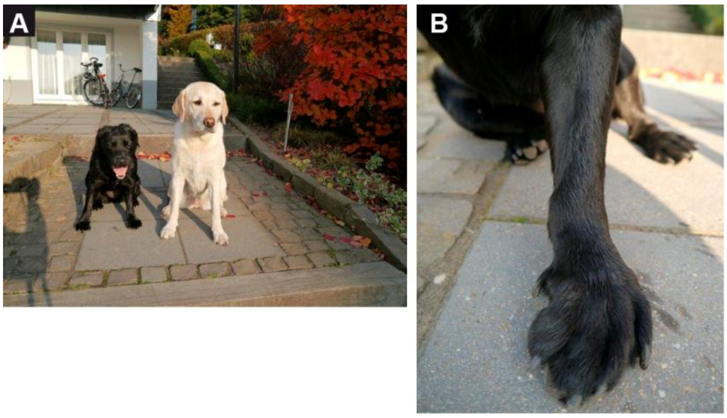

Figure S1. Photographs of a Labrador Retriever with very short legs and deformed paws that was originally considered to be a case in this study. Subsequently, it turned out that this dog did not carry the associated haplotype on CFA 12. Thus, we assume that the phenotype of this dog, which is more severe than in the other cases in our study, is caused by a different genetic or environmental effect (phenocopy). (A) The affected black dog next to a non-affected yellow Labrador Retriever. (B) Details of the deformed front left paw.
