# Supplementary figures and images for: A COL11A2 Mutation in Labrador Retrievers with Mild Disproportionate Dwarfism
Source: PLoS One. 2013 Mar 20;8(3):e60149. doi: 10.1371/journal.pone.0060149 (PMC3603880; doi:10.1371/journal.pone.0060149)

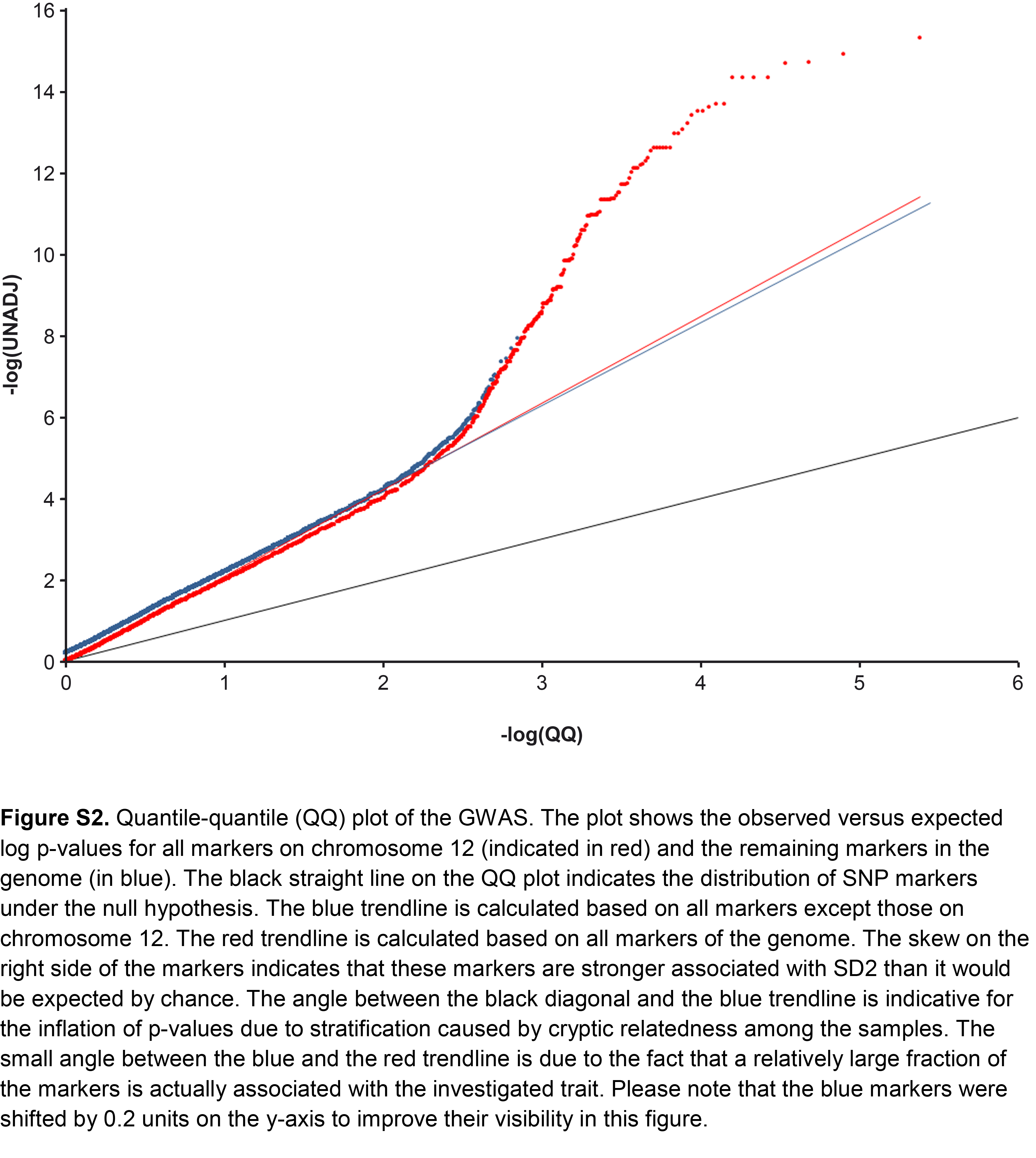

Supplement: Figure S2 — Quantile-quantile (QQ) plot of the GWAS. (TIF) [file pone.0060149.s002.tif]
